# Who Is Who: An Anomalous Predator-Prey Role Exchange between Cyprinids and Perch

**DOI:** 10.1371/journal.pone.0156430

**Published:** 2016-06-08

**Authors:** Lukáš Vejřík, Ivana Matějíčková, Jaromír Seďa, Petr Blabolil, Tomáš Jůza, Mojmír Vašek, Daniel Ricard, Josef Matěna, Jaroslava Frouzová, Jan Kubečka, Milan Říha, Martin Čech

**Affiliations:** 1 Biology Centre of the Czech Academy of Sciences, v.v.i., Institute of Hydrobiology, Na Sádkách 7, 37005, České Budějovice, Czech Republic; 2 Faculty of Science, University of South Bohemia in České Budějovice, Branišovská 31, 37005, České Budějovice, Czech Republic; University of Hyogo, JAPAN

## Abstract

Piscivory in cyprinids (Cyprinidae) is extremely rare. Specifically, common bream (*Abramis brama*) and common carp (*Cyprinus carpio*) are zooplanktivorous fish in deep lentic waters. Nevertheless, we observed predation by these two cyprinids under natural conditions in the Vír Reservoir, Czech Republic. We conducted diet analysis for cyprinids caught by trawling and gillnets and the large amount of young-of-the-year (YOY) perch (*Perca fluviatilis*), with sizes of 37–52 mm standard length, were found in their digestive tracts. In 2010, a large amount of YOY perch caused a significant decrease in *Daphnia* spp. size and abundance in the reservoir. Hence, a food deficit was induced for the cyprinids, apparent also from the poor nutritional condition of common bream which was much worse than the condition of those in similar reservoirs. Common carp and common bream shifted to forced piscivory, and they utilized the YOY perch as an alternative food source. In contrast, smaller species, such as roach (*Rutilus rutilus*) and bleak (*Alburnus alburnus*), widely utilized planktonic cyanobacteria. In the following year, YOY perch occurred in significantly lower numbers and conversely, *Daphnia* spp. size and abundance were significantly higher. The forced piscivory was not observed. Our results indicate a switch to forced piscivory by cyprinids, which was caused by a shortage of their natural food source. Moreover, this phenomenon presents an effective mechanism for reduction in the numbers of YOY perch, ensuring the stability of the ecosystem.

## Introduction

In freshwater ecosystems, two basic interactions between perch (*Perca fluviatilis*) and cyprinids (Cyprinidae) are commonly described. The first is the predator-prey interaction where adult perch prey on cyprinids [1–3]. In addition to the predatory role of adults, young-of-the year (YOY) perch have also been documented to prey on YOY common bream (*Abramis brama*) [4], although zooplankton is their dominant prey item [5–8]. The second interaction is the interspecific competition. Although cyprinids are primarily omnivores [9,10], they are usually zooplanktivores in deep lentic waters [11,12]. Therefore, zooplankton, namely *Daphnia* spp., are an essential food source for both perch and cyprinids [3,12–15]. The competition for *Daphnia* spp. is indirectly emphasized by fish kairomones produced by abundant YOY perch. The presence of kairomones induces the shift of *Daphnia* spp. into deeper pelagic water layers, resulting in them being inaccessible to the fish [7,16–18].

Usually, extremely large YOY perch populations are effectively reduced by intercohortal cannibalism [19–23] or by other typical predators, such as pike (*Esox lucius*) and pikeperch (*Sander lucioperca*) [24–26]. A less studied mechanism for reducing YOY perch population is auto-reduction. In this case, the strong predation pressure on zooplankton causes depletion of the food source, and YOY perch consequently die from starvation [27]. No information about reduction of YOY perch by cyprinids has been previously presented.

Although cyprinids are known for their diet plasticity [9–11,28], piscivorous feeding is very rare. Except for asp (*Aspius aspius*) [29] and the *Labeobarbus* species flock [30], piscivory in cyprinids has only been observed to limited extent in common carp (*Cyprinus carpio*) [31,32] and rudd (*Scardinius erythrophthalmus*) [33–35].

Our study describes a reversal of the typical predator-prey interaction between perch and cyprinids. Specifically, it is focused on (a) the impact of an extremely numerous YOY perch cohort on zooplankton and the effect this has on the population of cyprinids, (b) the highest rate of cyprinids piscivory observed to date and the first proof of piscivory by the common bream, and (c) a general discussion of the significance of piscivorous feeding by omnivorous cyprinids.

## Methods

### Study area

**Fig 1:**
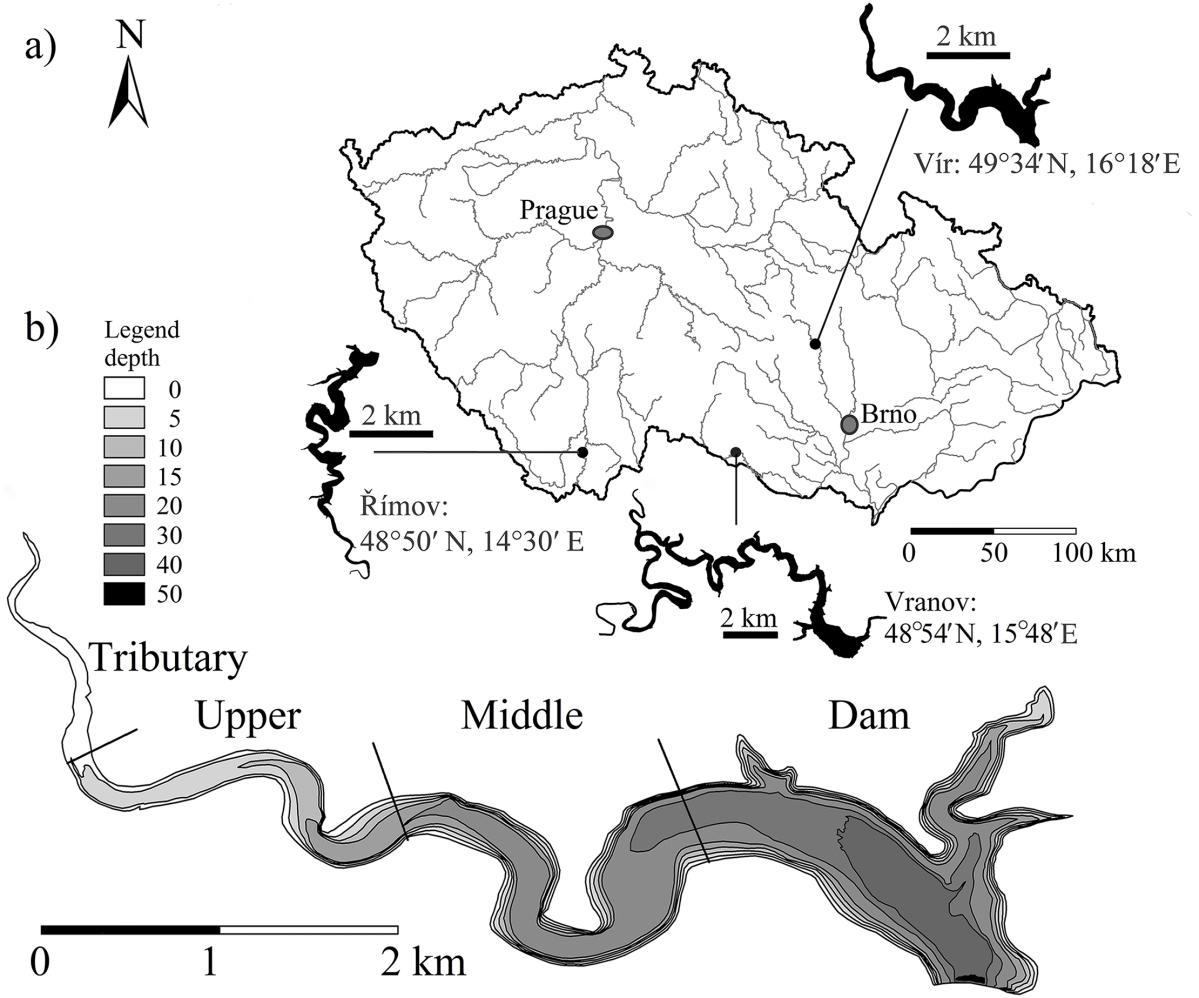
A map showing the location of the Vír, Vranov and Římov Reservoirs in the Czech Republic (a) and a detailed view of the bathymetric map of the Vír Reservoir (b). In (b), 0 corresponds to the surface level in late July 2010. The legend shows contour lines with relevant depths. The sampling design of the Vír Reservoir consisted of four sections (Tributary, Upper, Middle and Dam) along its longitudinal gradient.

The main part of the present study was conducted in the canyon-shaped Vír Reservoir located in the eastern part of the Czech Republic (49°34′ N; 16°18′ E; Fig 1). The maximum surface altitude is 464 m a.s.l. The reservoir has a surface area of c. 224 ha, a length of 9.3 km and total water volume of c. 56 × 10^6^ m^3^. Maximum and mean depths are 64 m and 25 m, respectively. It is characterized as eutrophic, and since 1992, cyanobacterial blooms have increasingly occurred, dominated mainly by *Microcystis* sp. [8].

As a comparison, two reference reservoirs (Vranov and Římov) were used for the length-mass relationship in common bream and for the size and abundance of *Daphnia* spp. The eutrophic Vranov Reservoir is a canyon-shaped reservoir located in the southeastern part of the Czech Republic (48° 54′ N, 15° 48′ E; 352 m a.s.l.). The reservoir has a surface area of 761 ha, a length of 28 km and total water volume of c. 133 × 10^6^ m^3^. The maximum and mean depths are 45 m and 15 m, respectively [36]. The meso- to eutrophic Římov Reservoir is a narrow, canyon-shaped reservoir located in the southern part of the Czech Republic (48°50′ N, 14°30′ E; 472 m a.s.l.; Fig 1). The reservoir has a surface area of 210 ha, a length of 9 km and total water volume c. 34 × 10^6^ m^3^. The maximum and mean depths are 45 m and 16 m, respectively [11].

### Abiotic factors

In the Vír Reservoir, water temperature and transparency were measured 10:00–14:00 (day sampling) in conjunction with the zooplankton sampling. To distinguish the epi-, meta- and hypolimnion, temperatures were measured at 1-m intervals throughout the entire water column of a dam section of the reservoir (to a maximum depth of 55 m, or when the probe reached the bottom) using a calibrated YSI 556 MPS probe. A Secchi disk was used to measure the water transparency.

### Trawl sampling

In the Vír Reservoir, trawling for adult fish was conducted during the night of July 27, 2010 (22:00–03:00). A pelagic trawl with a mouth opening of 13×8 m was used to sample adult fish. The trawl was towed approximately 100 m behind the research vessel, usually for 10–25 minutes, at speeds of 1.1–1.5 m s^-1^. For technical details, see Vejřík *et al*. [8]. The reservoir was divided into four approximately equidistant sections along its longitudinal axis, the dam, middle, upper and tributary sections; each section was approximately 2 km in length (Fig 1). Five tows were conducted in the dam and middle sections of the reservoir. It was not possible to conduct tows in the open water layers of the upper and tributary reservoir sections due to insufficient depth in these sections. All fish from each trawl tow were immediately anaesthetized by a lethal dose of tricaine methanesulfonate (MS–222, Sigma Aldrich Co.). The fish were identified to the species level, counted, measured (SL–standard length, and TL–total length) and weighed. The adult trawling was not repeated in 2011 due to incredibly demanding and time-consuming sampling campaign involving financial and manpower limitations. Trawling for YOY fish was carried out on July 26 (night) and July 27 (day), 2010, and on July 31 (night) and August 1 (day), 2011. A pelagic, fixed-frame fry trawl with a mouth opening of 3×3 m was used to sample the YOY fish. The trawl was towed approximately 100 m behind the research vessel, usually for 10 minutes, at speeds of 0.8–1.1 m s^-1^. A total of 63 tows were conducted. For technical details, see Vejřík *et al*. [8]. All of the juvenile fish from each trawl tow were immediately anaesthetized by a lethal dose of MS–222 and subsequently preserved in 4% formaldehyde. In the laboratory, the fish were identified to the species level and counted. SL was measured on 1,000 individual YOY perch from both years. The catch was expressed as the number of fish per 100 m^3^ of water volume sampled.

### Pelagic gillnet sampling

In addition to adult trawling, pelagic gillnets were used to sample adult cyprinids in the open water of the Vír Reservoir in 2010 and 2011. They were also used to sample the common bream used as reference from the Římov Reservoir in 2010 and the Vranov Reservoir in 2011.

Gillnets were placed from the surface to a depth of 4.5 m and between 5 and 9.5 m. The design of the pelagic gillnets followed the European standard [37] and was supplemented by gillnets with larger mesh sizes according to [38]. Gillnets (n = 3 per section for both types) were set one hour prior to sunset and collected one hour after sunrise to cover the highest peaks in fish activity [39] in the dam, middle and upper reservoir sections on July 28 in 2010 and August 2 in 2011 in the Vír Reservoir, on July 27, 2011 in the Vranov Reservoir and on August 10, 2010 in the Římov Reservoir. In the upper section, gillnets were not set at 5–9.5 m depth due to shallow water. A total of 18 gillnets were set to the 0–4.5 m depth and 12 to the 5–9.5 m depth. The fish used for gut content analysis were taken out three hours after installation, and the remaining fish were taken out at the end of installation. All fish from the gillnets were immediately anaesthetized using a lethal dose of MS–222, identified to the species level, counted, measured (SL, TL) and weighed.

### Zooplankton sampling

Zooplankton was sampled near the dam during the daytime on June 9 and July 27, 2010 and on June 3 and July 30, 2011 in the Vír Reservoir. In the reference reservoirs, zooplankton was sampled on June 6 and July 22, 2011 in the Vranov Reservoir and on June 7 and July 26, 2010 in the Římov Reservoir. Nighttime sampling was not conducted, as previous studies have confirmed no apparent diurnal vertical migration by zooplankton in manmade reservoirs [40] in contrast to results from natural lakes [7].Two different closing nets were used because the abundance of zooplankton differed by more than one order of magnitude between the upper and deep water layers. A net with an opening of 24 cm (diameter) was used for the epilimnion, and a net with an opening of 40 cm was used for the deeper water layers. Both nets had a 170-μm mesh size. The zooplankton samples were immediately preserved in 4% formaldehyde. Zooplankton samples were collected in four independent replications from the epi-, meta- and hypolimnion. Zooplankton specimens were identified to species level according to [41,42] using a microscope (Olympus CX40), and counted according to [43]. Each zooplankton sample was diluted so that subsampling by a wide mouth pipette resulted in c. 200–250 individuals. Four subsamples were counted separately in a Sedgewick-Rafter counting chamber. When abundances in the bulk sample were low (usually from the deep water strata), the whole sample was processed. Abundance was calculated as an average per 1 L of water within each 3 m thick depth layer. Only the abundance of *Daphnia* spp. was used in this study because it dominates the diet of the fish. The *Daphnia* communities in the Vír and Vranov Reservoirs were identified as belonging to the *Daphnia longispina* complex and those in the Římov Reservoir to the *Daphnia galeata*. For more details, see [44,45]. A subsample of 300 individuals from each layer was digitally photographed under the microscope for subsequent measurement of carapace size. The minimum size for individuals capable of reproduction was determined to be 0.95 mm, according to [44].

### Diet analysis

Diet analysis was conducted for randomly chosen individual common bream. Specifically, 100 individuals from the adult trawling and 50 individuals from the pelagic gillnets in 2010 and all individuals from the pelagic gillnets in 2011 were dissected. Diet analysis was conducted for all common carp, roach (*Rutilus rutilus*) and bleak (*Alburnus alburnus*) captured by the adult trawl in 2010 and for 50 randomly chosen individual bleak and 50 randomly chosen individual roach captured by pelagic gillnets in 2011.

The digestive tracts of cyprinids were dissected and preserved in a 10% formaldehyde solution for subsequent laboratory analysis. All three intestinal loops were examined. The percent composition of the diet by volume was visually estimated and the state of the food remains was evaluated (well-preserved, slightly digested, highly digested). Five categories of food were distinguished: YOY fish, zooplankton, insects or zoobenthos, planktonic cyanobacteria (mainly *Microcystis* sp.) and detritus. Species and size were directly determined in the case of well-preserved fish collected from the digestive tracts. The more digested fish from the digestive tracts were identified to the species level and size using a reference collection with diagnostic bones of each potential prey species [46,47].

The vertebrate work was approved by the Ethics Committee of the Czech Academy of Sciences. All sampling procedures and experimental manipulations were approved by the Czech Academy of Sciences, Morava River Authority and the Environmental Department of the Municipal Authority of the Town of Brno. The field study did not involve endangered or protected species.

### Statistical analysis

The nonparametric Kruskal-Wallis test was used to compare the differences between YOY perch length found in common carp and common bream digestive tracts and between the YOY perch length in the Vír Reservoir in 2010. The same test was used to compare the differences of YOY perch abundances between 2010 and 2011.

One-way ANOVA was used to test the differences of YOY perch length between 2010 and 2011. The same test was used for one set of variables concerning the zooplankton data. A chi-square test (χ^2^) was used to compare the contribution of piscivorous common bream in the population between 2010 and 2011. Further, the same test was used to compare the contribution of zooplankton and cyanobacteria in the roach and bleak diets between 2010 and 2011. A chi-square test (χ^2^) was also used to compare the ratio of zooplankton abundance within the pelagic water layers between June 2010 and 2011 and between July 2010 and 2011.

A generalized linear model with a log link function was used to fit and compare the length-mass relationships of 188 common bream with SL larger than 260 mm (hereafter referred to as minimum length, min SL) obtained by sampling the three reservoirs (Vír, Vranov and Římov) in 2010 and 2011. Because of the length restriction (common bream > 260 mm SL) the minimum size was set to zero. Therefore, intercepts and exponents for each model could be easily compared. Factorial ANOVA was used to test for differences in zooplankton sizes. Tukey’s HSD post-hoc test was used to compare the differences in zooplankton sizes within the pelagic water layers (epi-, meta-, and hypolimnion).

Fulton’s condition factor (*FCF*) [48] was calculated as:

$$FCF\;=\;\frac{m}{TL^{3}}\times 100$$

where *m* stands for fish mass (g) and *TL* for fish total length (cm).

The prey-to-predator length ratio (*PPR*) was calculated as:

$$\;PPR\;=\;\frac{SL_{Py}}{SL_{Pr}}$$

where *SL*_*Py*_ stands for SL of prey and *SL*_*Pr*_ for SL of a predator.

All statistical tests were performed in the R environment for statistical computing (version 3.2.2) [49].

## Results

### YOY fish

**Fig 2:**
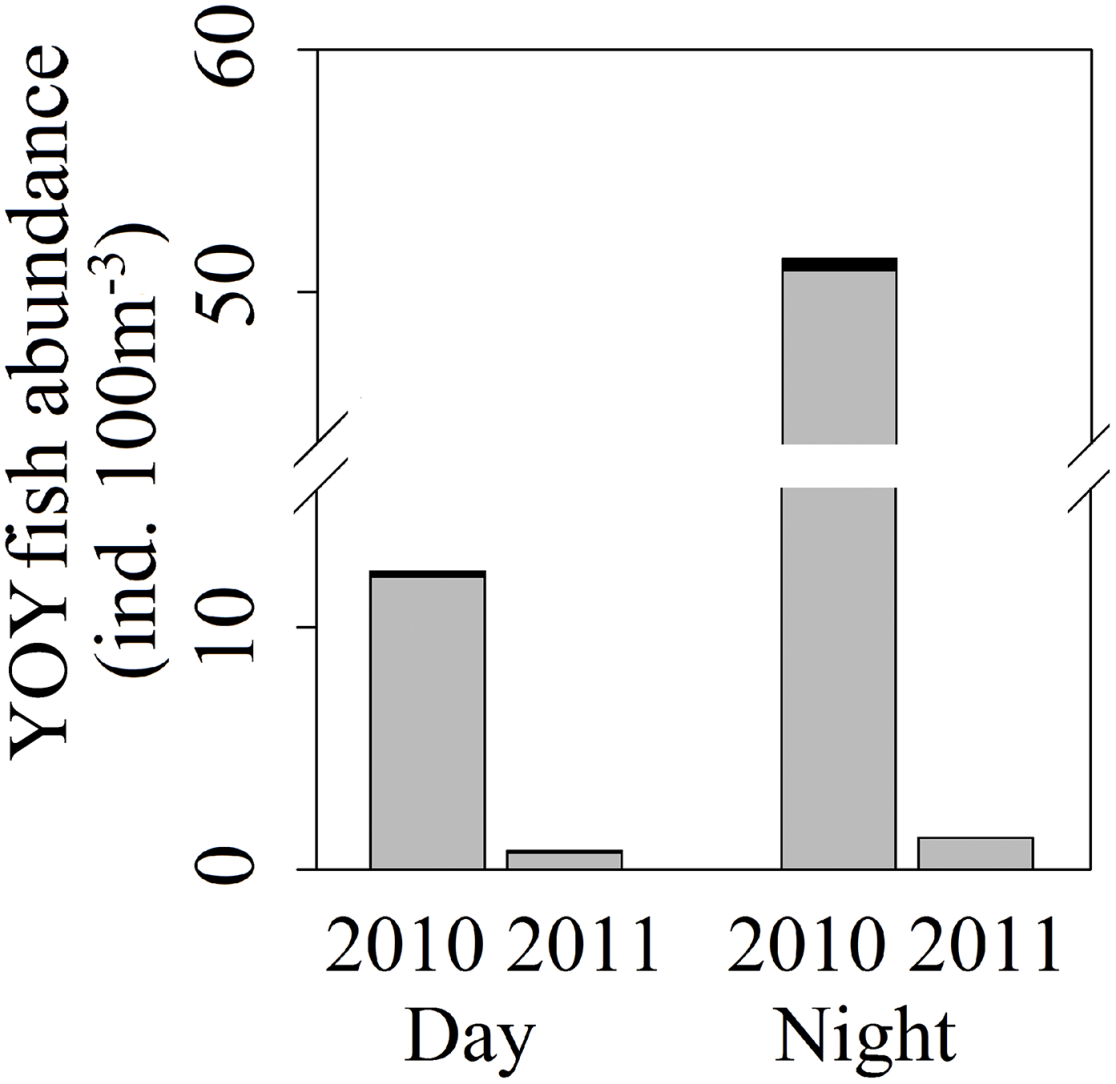
Abundance of YOY fish in pelagic water layer of the Vír Reservoir in 2010 and 2011. Mean abundance of YOY perch (grey colour)and other YOY fish (black colour)according to the fry trawl catches (Number of tows: 63, sampled water volume: 337.413 m^3^).

During the last week of July in 2010 and 2011, 45,047 YOY fish of seven species were caught by fry trawl in the Vír Reservoir: perch, pikeperch, common bream, bleak, roach, ruffe (*Gymnocephalus cernuus*) and European catfish (*Silurus glanis*). Percids made up 99% of the catch in both years and in all sampled reservoir sections at all depths, except at the 0–3 m depth in the upper section in 2011, where cyprinids were predominant. The most abundant species was perch followed by pikeperch. According to the averaged values from all tows, perch composed 98% (12.1 ind. 100 m^-3^) of daytime and 99% (50.9 ind. 100 m^-3^) of nighttime catches in 2010 and 84% (0.7 ind. 100 m^-3^) of daytime and 99% (1.32 ind. 100 m^-3^) of nighttime catches in 2011 (Fig 2). Significant decrease of YOY perch abundance between the years 2010 and 2011 was observed. Compared to situation in 2010, the mean daytime and nighttime YOY perch densities in 2011 declined by 94% and 98%, respectively. Both differences were statistically significant (*H*_2,34_ = 4.7 *P* = 0,03 for daytime catches and *H*_2,29_ = 6.2 *P* = 0.01 for nighttime catches). SL (mean ± SD) of YOY perch were 44.2 ± 3.3 mm in 2010 and 37.5 ± 4.1 mm in 2011. The length differences was statistically significant (*F*_1,2000_ = 1622, *P* < 0.001).

### Adult fish and diet

During the adult night trawling in 2010, 1,042 fish of 11 species were caught in the Vír Reservoir. Four species of zooplanktivores were caught: common bream, common carp, bleak and roach. Common bream dominated the catch, making up in excess of 85% of the captured fish (886 individuals), followed by common carp (3.6%, 37 individuals), bleak (3%, 31 individuals) and roach (2%, 21 individuals). Typical fish predators, such as European catfish, European eel (*Anguilla anguilla*), pikeperch, asp and perch composed only 6.4% of the catch (a total of 67 individuals).

Another 1,082 individual fish older than YOY were caught by pelagic gillnets in the Vír Reservoir. Four species of zooplanktivores and benthivores were caught: common bream, bleak, roach and common carp. Bleak composed 64.1% of the total catch (693 individuals). Common bream was the second most common species at 15.1% of the total catch (164 individuals), roach composed 9.1% of the total catch (99 individuals) and common carp composed only 0.4% of the total catch (4 individuals). Typical predators such as pikeperch, asp and perch composed 11.3% of the total catch (122 individuals).

**Fig 3:**
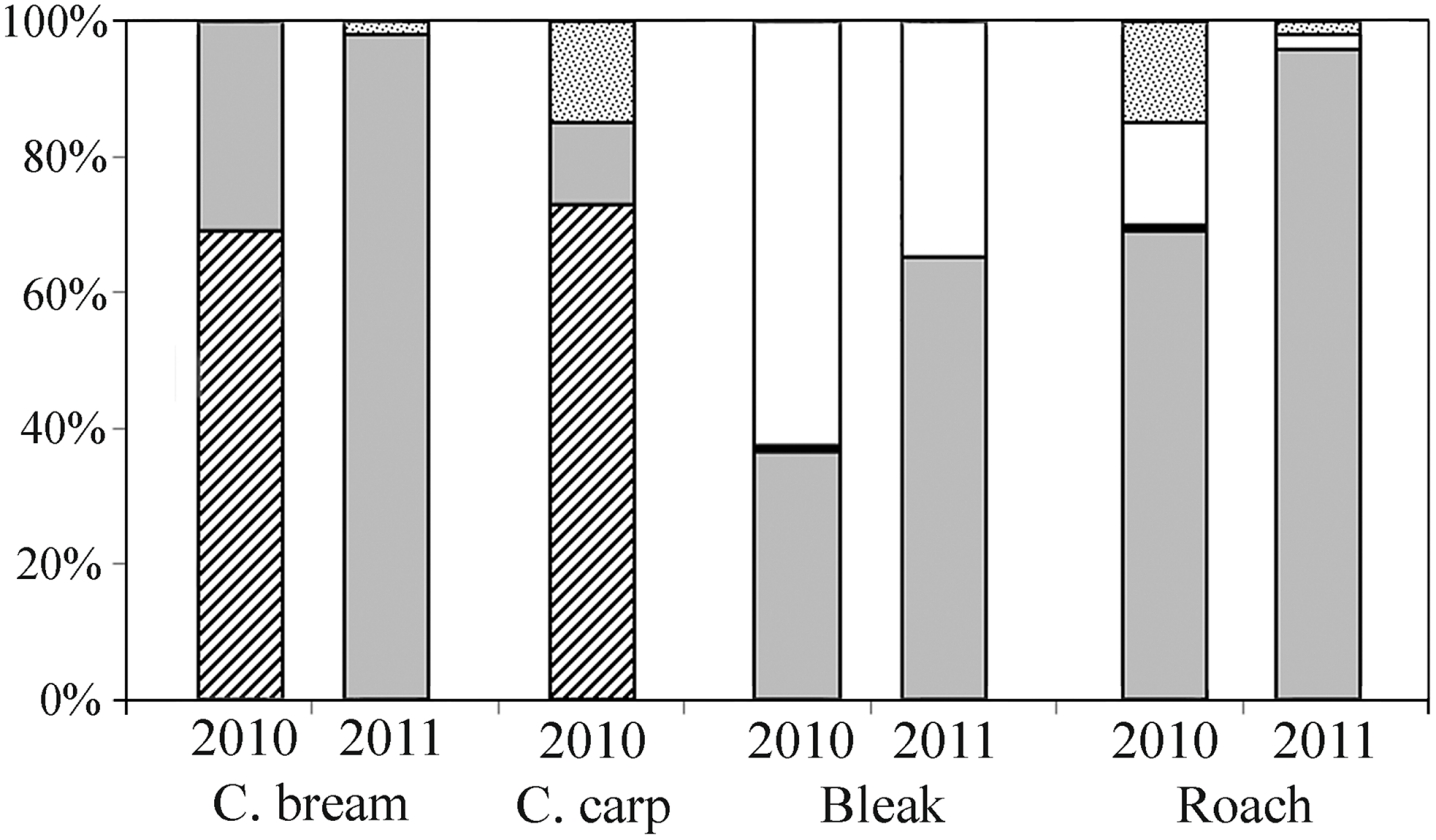
The gut contents of cyprinids from the Vír Reservoir in 2010 and 2011. The caught cyprinids were: common bream (*Abramis brama*), common carp (*Cyprinus carpio*), bleak (*Alburnus alburnus*) and roach (*Rutilus rutilus*). No common carp were caught in 2011. The gut content was divided into five categories: YOY fish (dashed), Zooplankton (grey), Insect and Zoobenthos (black), Planktonic Cyanobacteria (white), and Detritus (dotted).

In adult trawl catches in 2010, YOY fish were found in the diet of 72 individual common bream (48%) out of the 150 analyzed individuals within the SL range of 255 and 390 mm (mean ± SD: 304 ± 31.6 mm). In addition, YOY fish were found in 22 individual common carp (60%) out of the 37 individuals within the SL range of 155 and 285 mm (290 ± 26.7 mm). Neither roach (SL 100–260 mm, 180 ± 18.4 mm) nor bleak (SL 85–130 mm, 113 ± 10.1 mm) contained fish in their digestive tracts (Fig 3). All YOY fish found in common bream and common carp digestive tracts were identified as perch. YOY perch comprised 25–100% (mean 69%) and 33–100% (mean 73%) of the gut content in piscivorous common bream and common carp, respectively. The rest of the gut content in both fish species was composed primarily of zooplankton, with detritus also found in common carp. The roach diet was composed of zooplankton, planktonic cyanobacteria (*Microcystis* sp.) and detritus. The bleak diet consisted of planktonic cyanobacteria, zooplankton and terrestrial insects. Further, the diet analysis provided for 50 individual common bream and 4 individuals of common carp from gillnets revealed YOY fish in the diet of 25 individual common bream (50%) within the SL range of 230 and 385 mm (mean ± SD: 301 ± 25.6 mm). In common carp, YOY fish were found in 3 individual common carp (75%) within the SL range of 200 and 280 mm (227 ± 35.9 mm) (Fig 3). The contribution of piscivorous common bream and common carp in trawl catches and gillnet gatches did not statistically differ in 2010 (χ^2^ = 0.46, *P* = 0.49, and χ^2^ = 3.6, *P* = 0.06).

In 2011, 750 individual fish older than YOY were caught by pelagic gillnets in the Vír Reservoir. Three species of zooplanktivores and benthivores were caught: common bream, bleak and roach. Common carp were not caught in 2011. Bleak composed 70% of the total catch (520 individuals). Common bream was the second most common species at 11% of the total catch (85 individuals) and roach composed 10% of the total catch (78 individuals). Typical predators such as pikeperch, asp and perch composed 9% of the total catch (67 individuals). In 2011, no YOY perch were found in the digestive tract of common bream.

YOY fish found in the first intestinal loop of common bream and common carp in 2010 were well-preserved and easily identifiable and measurable. The preservation decreased in the second intestinal loop. White, highly digested emulsion was mostly found in the third loop. Ninety-four individual YOY perch from common bream digestive tracts could be measured. Their SL was between 37 and 52 mm (mean ± SD: 44.7 ± 2.8 mm). Although the size of YOY perch in common bream digestive tracts was slightly larger than in the overall reservoir, the difference was insignificant (*H*_2,195_ = 2.27, *P* ˃ 0.1). Thirty-five individual YOY perch from common carp digestive tracts could be measured. Their SL was between 38 and 50 mm (44.3 ± 3.5 mm). The size difference between YOY perch in common carp digestive tracts and in the overall reservoir was insignificant (*H*_2,136_ = 0.427, *P* ˃ 0.5). Prey-to-predator length ratios (PPR) ranged from 0.10 to 0.17 (mean: 0.13) for bream and from 0.14 to 0.24 (mean: 0.19) for common carp.

There was a significant decrease in the contribution of piscivorous individual common bream in the population between 2010 and 2011 (χ^2^ = 85.4, *P* < 0.001). In 2011, no YOY perch were found in digestive tracts of analyzed fish. The diet of common bream (SL 240–380 mm, mean ± SD: 311 ± 21.6 mm) was composed of zooplankton (98%) and detritus (2%). Zooplankton was also the main component of the diet for roach (SL 85–255 mm, 165 ± 21.1 mm). In the roach diet, the contribution of zooplankton significantly increased (χ^2^ = 189.8, *P* < 0.001), and in contrast, the contribution of planktonic cyanobacteria significantly decreased (χ^2^ = 86.2, *P* < 0.001) between 2010 and 2011. In the bleak diet (90–135 mm, 120 ± 11.4 mm), similar trend of increase of zooplankton (χ^2^ = 26.1, *P* < 0.001) and decrease of planktonic cyanobacteria (χ^2^ = 36.1, *P* < 0.001) was observed between 2010 and 2011.

### Bream condition

**Fig 4:**
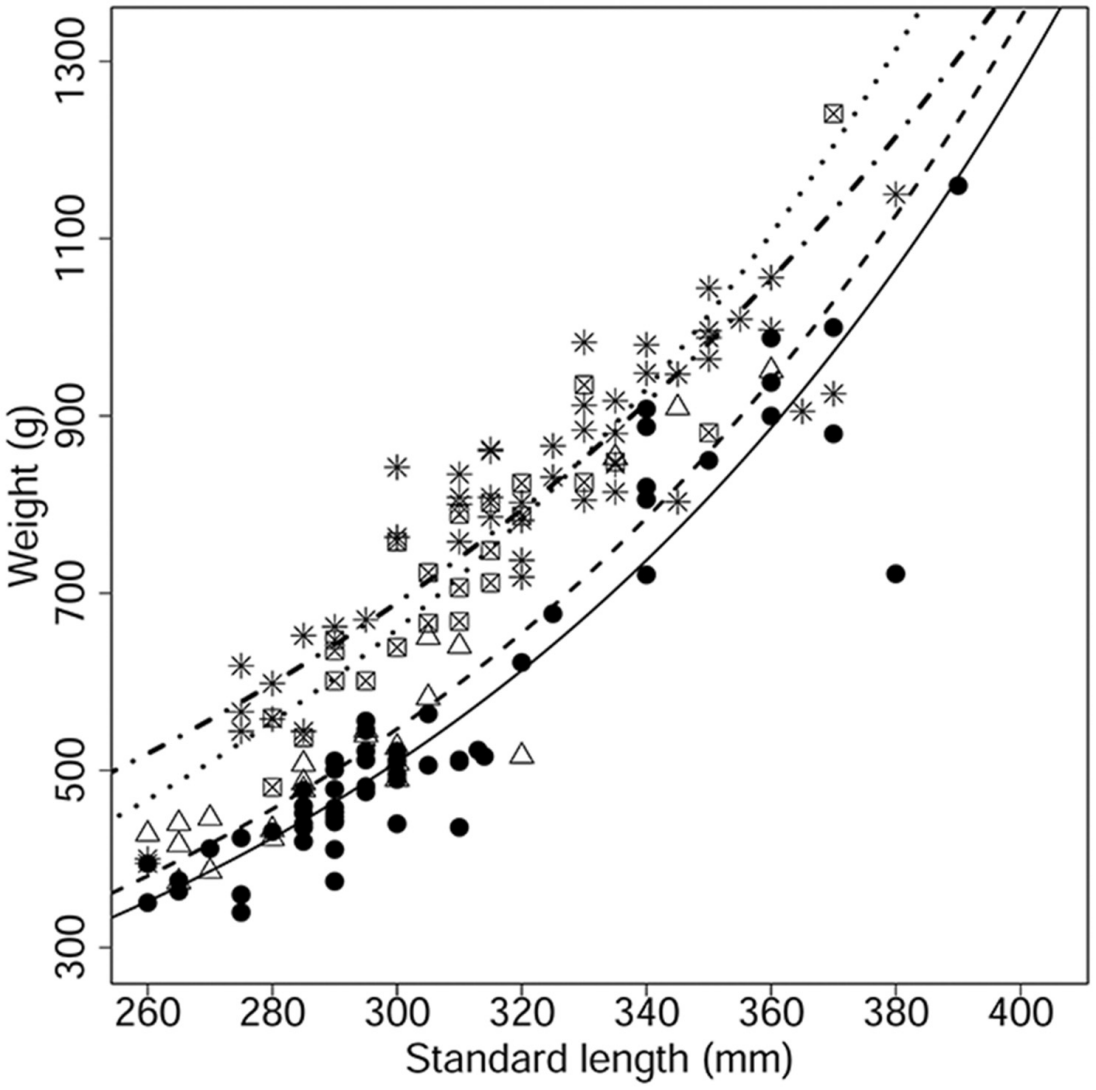
Comparison of length-weight relationships of common bream (*Abramis brama*). Data of 179 individuals with standard length ˃ 260 mm were obtained by sampling from three reservoirs in 2010 and 2011. The comparison was performed by generalized linear model with a log link function. Legend: Vír Reservoir 2010 (designated with dots and solid line, y = 5.864 × e ^0.0092^), Vír Reservoir 2011 (triangles and dashed line, y = 5.942 × e ^0.0090^); Vranov Reservoir 2011 (crossed square and dotted line, y = 6.146 × e ^0.0086^) and Římov Reservoir 2010 (asterisk and dash-dotted line, y = 6.252 × e ^0.0071^).

The poor nutritional condition of bream in the Vír Reservoir in 2010 was apparent from their length-mass relationship in comparison to the bream from the Vír Reservoir in 2011 and to those from the Římov and Vranov Reservoirs (Fig 4). The comparison of length-mass relationships by generalized linear models with log link functions revealed a significant difference between the intercepts for the Vír Reservoir bream from 2010 and those for the bream from the other sampling campaigns (*P* < 0.05). However, the changes in exponents were similar (*P* > 0.05), with the exception of those from the Římov Reservoir analysis (*P* < 0.001). This means that the growth curves of fish were similar in all reservoirs, but the mass at the same length was different. The lowest length-mass relationship was found for common bream from the Vír Reservoir in 2010. The following year the curve was higher, but it was still below the reference reservoirs (Fig 4).

Means of the Fulton’s condition factor for common bream in particular reservoirs were as follows: *FCF*_(Vír 2010)_ = 0.975 ± 0.091 (mean ± SD), *FCF*_(Římov 2010)_ = 1.251 ± 0.140 (mean ± SD), *FCF*_(Vranov 2011)_ = 1.243 ± 0.065 (mean ± SD) and *FCF*_(Vír 2011)_ = 1.085 ± 0.156 (mean ± SD). Both analyses (results of length-mass relationships and Fulton’s condition factors) indicate that the worst bream condition was found in 2010 in the Vír Reservoir and that there was an apparent improvement in 2011.

### Zooplankton

The *Daphnia* community in the dam section of the Vír and Vranov Reservoirs was formed by the *Daphnia longispina* species complex, which was dominated by *D*. *longispina*, *D*. *galeata* and their hybrids. All specimens are subsequently referred to as *D*. *longispina* cp. The presence of *D*. *cucullata* was recorded in 2010 in the dam section of the Vír Reservoir. The *Daphnia* community in the dam section of the Římov Reservoir was formed by the *Daphnia galeata*.

**Fig 5:**
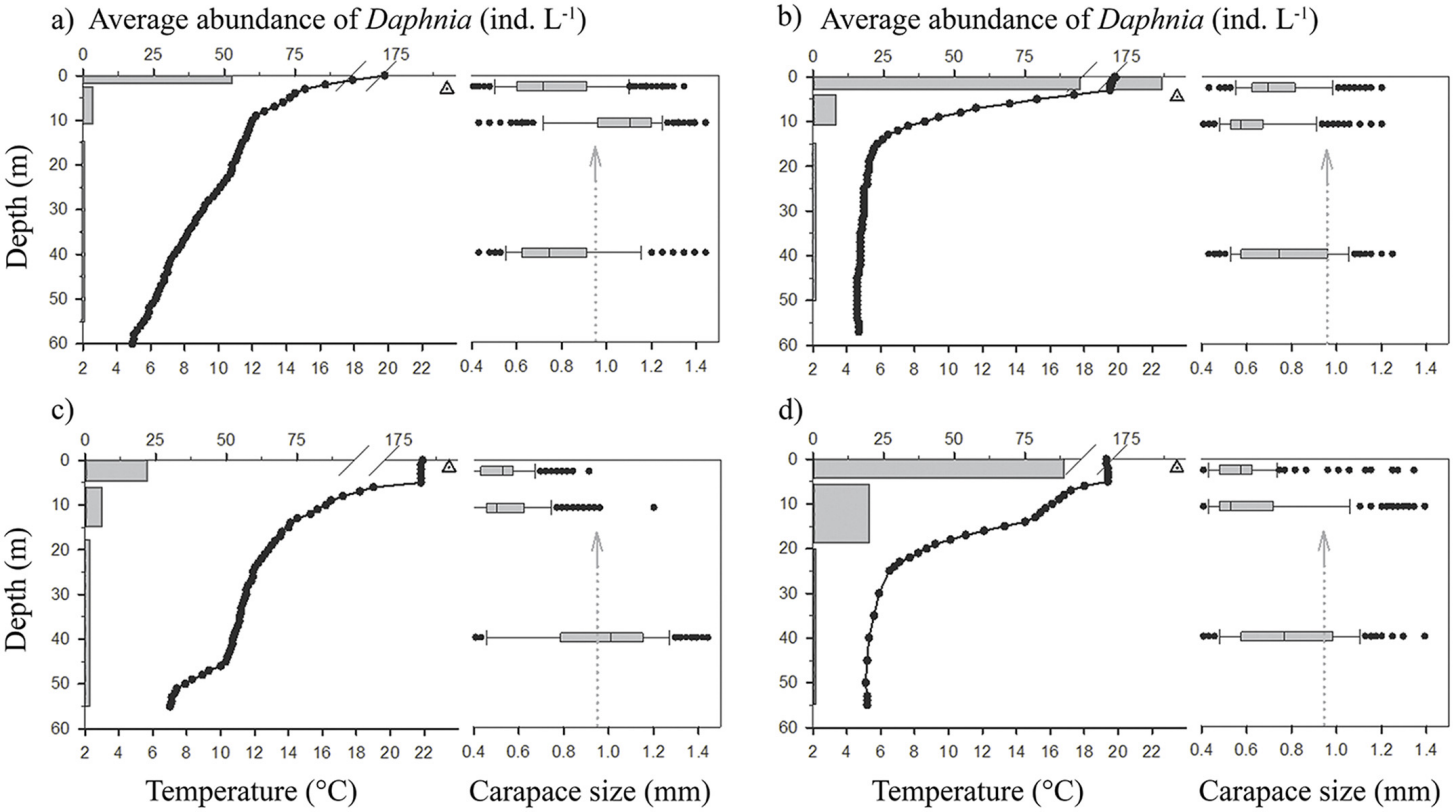
Vertical profile of abundance (horizontal bars for epi-, meta- and hypolimnion) and carapace size (box and whisker plots) of *D*. *longispina* cp. Data were obtained in the dam section of the Vír Reservoir in 2010 for a) June and c) July, and in 2011 for b) June and d) July. Black lines with black dots show the vertical temperature profiles. Box and whiskers plots: median values, upper and lower quartiles (boxes), maximum and minimum values (whiskers), and all outliers (dots) are shown. Arrows with dotted line indicate the minimum carapace size needed for reproduction according to Petrusek *et al*. [44]. The triangle in the center indicates water transparency measured by Secchi disk.

Both the abundance and size of *D*. *longispina* cp in the Vír Reservoir differed among the depth layers and between the sampled months and years (Fig 5; Table 1). The carapace size in the upper layers (i.e., epi- and metalimnion) significantly decreased between June and July 2010 (epilimnion: *F*_1,608_ = 290, *P* < 0.001; metalimnion: *F*_1,604_ = 1,091, *P* < 0.001). In contrast, the size significantly increased in the hypolimnion (*F*_1,615_ = 59, *P* < 0.001). In 2011, the differences in the size of *D*. *longispina* cp in particular pelagic layers were less apparent between June and July. A significant decrease in size was noticed only in epilimnion (*F*_1,576_ = 84, *P* < 0.001; Fig 5; Table 1). In the reference reservoirs, a decrease in the size of *Daphnia* spp. in the epi- and metalimnion was observed also in the Vranov Reservoir between June and July, and an increase in size was observed in the Římov Reservoir. Nevertheless, the sizes in both reference reservoirs were significantly larger than in the Vír Reservoir in 2010 (*F*_2,1800_ = 1225, *P* < 0.001). In addition, the decrease in size in *Daphnia* spp. from the Vranov Reservoir was not as apparent as in those from the Vír Reservoir in 2010 (Table 1). In the Vranov Reservoir, a slight increase in the size of *D*. *longispina* cp in the hypolimnion was observed, but again it was apparently less than the size change in the Vír Reservoir in 2010.

**Table 1:** Mean carapace size (± S.D.), mean abundance (± S.D.) and population percentage share (PPS; i.e., relative *Daphnia* abundance from the entire water column) of *D*. *longispina* complex in the Vír, Římov and Vranov Reservoirs.

Water	Parameter	Vír 2010		Vír 2011		Římov 2010		Vranov 2011	
layer		June	July	June	July	June	July	June	July
Epi-	Mean carapace size	0.75	**0.51**	0.73	0.59	0.66	0.75	0.9	0.6
	±SD (mm)	±0.21	**±0.12**	±0.18	±0.17	±0.20	±0.30	±0.28	±0.20
	Mean abundance	53	**21.89**	191.8	88.8	60	35	40.5	46.3
	±SD (ind. L^-1^)	±5.08	**±1.27**	±14.7	±8.95	±6.01	±2.5	±4.98	±5.05
	PPS (%)	74	**51**	87	55	74	72	86	96
Meta-	Mean carapace size	1.04	**0.54**	0.62	0.63	0.93	0.64	0.72	0.57
	±SD (mm)	±0.22	**±0.14**	±0.17	±0.25	±0.31	±0.26	±0.21	±0.16
	Mean abundance	3.65	**5.9**	8.23	19.8	7	1	4	1.43
	±SD (ind. L^-1^)	±0.53	**±0.56**	±1.32	±1.50	±1.65	±0.2	±0.54	±0.15
	PPS (%)	20	**25**	9	40	17	18	10	3
Hypo-	Mean carapace size	0.79	**0.95**	0.77	0.78	0.94	0.83	0.77	0.81
	±SD (mm)	±0.22	**±0.29**	±0.21	±0.24	±0.32	±0.27	±0.22	±0.27
	Mean abundance	0.25	**1.35**	0.68	1.3	0.5	0.4	0.66	0.17
	±SD (ind. L^-1^)	±0.06	**±0.24**	±0.19	±0.1	±0.16	±0.09	±0,2	±0.05
	PPS (%)	6	**24**	4	5	9	10	4	1

The data come from sampling of dam sections in June and July of given years for particular pelagic water layers (epi-, meta- and hypolimnion). The extreme period with high predation pressure of YOY perch is shown in bold.

*D*. *longispina* cp sizes in the hypolimnion of the Vír Reservoir in July of both years were significantly larger than in the epilimnion and metalimnion (*P* < 0.001 for all tests). However, the size differences were more obvious in July 2010. That year, almost no epilimnetic and metalimnetic individuals reached the minimum size needed for reproduction. In contrast, *D*. *longispina* cp sizes in the hypolimnion were larger, indicating that individuals capable of reproduction found a refuge in the deeper water layer (Fig 5). Such a distinct size difference of *Daphnia* spp. was not observed in either reference reservoirs (Table 1).

The population percentage share (i.e., relative *Daphnia* abundance from the entire water column) in the metalimnion and in the hypolimnion was higher in July than in June in both years. The highest population percentage share in the hypolimnion was observed in July 2010 (24%) and the lowest was observed in June 2011 (4%). The population percentage share of *D*. *longispina* cp within the water layers was significantly different between July 2010 and 2011 (χ^2^ = 24.4, *P* < 0.005). The difference was also significant between June 2010 and 2011 (χ^2^ = 9.0, *P* < 0.05) but less so than in the July comparison (Table 1). The population percentage share in the hypolimnion was markedly higher in the Vír Reservoir in July 2010 than in all other observed periods and reservoirs (Table 1).

## Discussion

Although Cyprinidae is one of the most species-rich (> 2000 species) and widespread freshwater fish family [50], piscivory is an extremely rare foraging strategy among this successful fish group [30]. Cyprinids are not well designed for piscivory because they lack teeth in their oral jaws, have a small slit-shaped pharyngeal cavity and lack a stomach with a low pH for digesting fish prey [30]. The only piscivorous cyprinids are the asp [29,51] and *Labeobarbus* species flock in Lake Tana, Ethiopia, where 8 of 15 species show signs of piscivory [30]. Facultative piscivory was rarely observed in introduced rudd and emerald shiner (*Notropis atherinoides*) in the Niagara River [34,35]. Furthermore, piscivory by rudd was probably observed in an experimental English lake with topmouth gudgeon (*Pseudorasbora parva*) as prey [33]. Piscivory was also observed for common carp on introduced topmouth gudgeon under experimental conditions [32] and for introduced common carp on small fish, probably tilapia (*Tilapia zillii*), in Naivasha Lake, Kenya [31]. Therefore, facultative piscivory has only been observed in introduced species or on introduced prey species where the natural species balance was affected in the ecosystem. In the situation presented here, perch, common bream and common carp are indigenous species in the Vír Reservoir of the Morava River catchment area [52].

Common bream and common carp diets consist mainly of zooplankton and zoobenthos in various proportions depending on the food availability and the location [11,14,29,53–55]. Hence, facultative piscivory by common bream and common carp in the Vír Reservoir is best explained as a reaction to the scarcity of invertebrate prey, specifically small sizes and low numbers of *D*. *longispina* cp in July 2010. During that piscivorous period, the average size of *D*. *longispina* cp was extremely small in the epilimnion (mean: 0.51 mm). Therefore, it did not reach the critical reproduction size. This was clear evidence of an extremely high predation pressure (*cf*. [44]). In contrast, *Daphnia* spp. sizes were apparently larger in the epilimnion layers of the Vír Reservoir in 2011 and of the reference reservoirs (Vranov, mean: 0.62 mm and Římov, mean: 0.75 mm). The presence of extremely high predation pressure in the Vír Reservoir in 2010 is supported by the occurrence of small-sized *Daphnia cucullata* in the dam section. This species commonly occurs only in the tributary section of reservoirs [44,56] where high fish biomass induces high predation pressure on zooplankton [57]. High predation pressure on zooplankton from YOY perch is also apparent from the gut content of other cyprinids. In 2010, a distinct share of planktonic cyanobacteria with a low nutritional value was found in bleak (mean: 60% of the gut content) and roach (mean: 15% of the gut content). In contrast, the share of planktonic cyanobacteria significantly decreased for both bleak (mean: 15% of the gut content) and roach (mean: 2% of the gut content) and the contribution of zooplankton increased in 2011. Utilization of cyanobacteria by cyprinids is typical for periods with significant food deficit [58,59].

In the Vír Reservoir in 2010, large individuals of *D*. *longispina* cp capable of reproduction occurred only in the hypolimnion refuge, which is avoided by cyprinids due to its low temperature, low oxygen concentration [8] and almost complete darkness [60]. This seeking of refuge by zooplankton in deep water layers due to intensive predation by YOY perch and the presence of YOY perch kairomones acting as a trigger is a well-known behavior [7,16,17]. Because *Daphnia* spp. were predominant in the YOY perch diet in the Vír Reservoir [8] and the number of YOY perch reached extreme values of 50.9 ind. 100 m^-3^ (mean) in 2010, the impact on zooplankton was substantial. The number of YOY perch was significantly lower in 2011 (1.32 ind. 100 m^-3^) causing lower predation pressure on zooplankton. Nevertheless, the number of YOY perch is commonly even lower, averaging 0.1 ind. 100 m^-3^ for nine other Czech reservoirs, including both reference reservoirs [61,62]. Accordingly, predation pressure of YOY perch on zooplankton must have been extremely high in the Vír Reservoir in 2010. Forced facultative piscivory by common bream and common carp was probably induced by extreme conditions, specifically a short-term but substantial absence of their planktonic food source. This resulted in a poor nutritional condition of common bream in the Vír Reservoir in 2010. Additionally, the length-mass relationship for common bream was significantly lower in 2010 than in 2011 in the Vír Reservoir and it was lower than for bream from either reference reservoirs. In 2011, no forced piscivory by common bream was observed. This poor nutritional state in 2010 was caused either by drastic traditional food limitation or by an inability to fully digest fish as an alternative and easily accessible food source, as was described by De Graaf *et al*. [30]. In this case, it was probably a combination of both factors.

Considering no significant difference in share of piscivorous common bream and common carp between trawl catches and gillnet catches in 2010, the gillnet catches were representative enough for the year 2011 However, the share of common carp in total gillnet catches was very low in 2010 and no individual was caught in 2011. From that reason, we can not confirm or disconfirm the piscivory by common carp in 2011. Absence of common carp in 2011 could be caused by low catch efficiency by gillnets towards this species (*cf*. trawl catches and gillnet catches in 2010) or decrease of common carp abundance in Vír Reservoir between 2010 and 2011 (*e*.*g*. by poaching).

The PPR of piscivorous common bream and common carp were on average 0.13 and 0.19, with maximum values of 0.17 and 0.24, respectively, which are very low values in general. Similarly low values were found for piscivorous *Labeobarbus* of Lake Tana (mean: 0.15, max: 0.25) [63]. These low values are likely due to the physiological limitations of cyprinids, which tend to be severely gape limited. In contrast, PPRs of non-cyprinid freshwater piscivores reach mean values from 0.25 to 0.40 and maximum values from 0.40 to 0.70 [64,65]. No statistical differences between length of YOY perch in the reservoir and in the digestive tract of common bream and common carp in 2010 indicated any diet preferences towards smaller or bigger individuals. Thus, significantly smaller YOY perch in the Vír Reservoir in 2011 was not a reason for absence of the piscivory. Due to gape limit of cyprinids (theoretical PPR for bream = 0.12), smaller YOY perch would be more likely expected to support piscivory. Therefore, the piscivory in 2010 was clearly induced by absence of primary food sources for common bream and common carp and not by simple preference for abundant fish prey.

The predation strategy of common bream and common carp remains as an unanswered question. According to Sibbing & Nagelkerke [66], some of Lake Tana’s piscivores of the *Labeobarbus* group likely use a variety of strategies (e.g., ambush hunters versus pursuit hunters). Based on the common bream and common carp mouth morphology, the strategy of a pelagic ambush hunter using velocity suction with protrusion is most probable [67]. This strategy has been demonstrated by *Labeobarbus megastoma* and *L*. *macrophthalmus* in Lake Tana [30]. Although the strategy of cyprinids is not fully clear, the lack of a significant difference between the length of YOY perch found in cyprinid digestive tracts and of those caught in the reservoir indicates that common bream and common carp did not prefer any particular sizes and that the prey selection likely depended on random encounters.

Despite of no information about common bream piscivory and scarce information about common carp piscivory in the scientific literature [31,32], this phenomenon may not be rare and may just be under studied. Common bream and common carp piscivory in the Vír Reservoir in 2010 occurred to a great extent and was apparently induced by extreme conditions caused by a large amount of YOY perch, as no piscivory was observed in 2011. Considering the numbers of traditional piscivorous fish (6.4% of trawl catch) relative to the numbers of common bream and common carp (88.6% of trawl catch) in the reservoir, we can conclude that piscivorous cyprinids may induce a much higher predation pressure on YOY perch than traditional piscivorous species.

The findings presented here highlight the key role of YOY perch in freshwater ecosystems. They can affect and change the behaviour of many species in fundamental ways. In the present case, YOY perch triggered (a) the shift of mature individuals of *D*. *longispina* cp into a hypolimnetic refuge and (b) the forced piscivory of cyprinids. Hence, our study under natural conditions illustrates that extreme situations require extreme solutions.

## Supporting Information

S1 Dataset Data file. Spreadsheet containing basic data required to reproduce the analyses, figures and table presented in the manuscript.
(XLS)
